# Editorial: Survival in Extreme Environments – Adaptation or Decompensation?, Volume II

**DOI:** 10.3389/fphys.2022.1106903

**Published:** 2022-12-16

**Authors:** Torkjel Tveita, Ingrid Eftedal, Sanjoy Deb

**Affiliations:** ^1^ UiT The Arctic University of Norway, Tromsø, Norway; ^2^ Department of Circulation and Medical Imaging, Faculty of Medicine and Health Sciences, Norwegian University of Science and Technology, Trondheim, SørTrøndelag, Norway; ^3^ Centre for Nutraceuticals, School of Life Sciences, University of Westminster, London, United Kingdom

**Keywords:** hypothermia, hypoxia, hypobaric and hyperbaric environments, Arctic environment, saturation diving, cerebral auto-regulation, cerebral compliance

## Introduction

Under normal environmental conditions human physiology describes the equilibrium of body homeostasis as a product of physiologic regulatory mechanisms, apparently resulting from a natural resistance to change from a pre-existing optimal biological condition. When humans experience extreme environments, like hyper/hypothermia, oxygen deprivation, or high-/low ambient pressures, the homeostatic equilibrium is challenged, and the victims may suffer from acute decompensation. Professional workers like divers, extraction industry workers, anglers and hunters, seamen, or people seeking these elements during leisure or recreational activity share this potential threat in case of accidents.

Therapeutic interventions after accidents in extreme environments was one key element in the invite for papers to this Research Topic. Another was the search for documentation for safe behavior to fight decompensation. Written guidelines for safe behavior and treatment of decompensated patient are seldom in place, and to write them we are in urgent need of knowledge. Due to obvious reasons, new knowledge of complex pathophysiologic mechanisms evoked by accidents under extreme conditions can often only be collected from experimental animal models.

## Hydration status in divers

Whilst few extreme exposures lend themselves conveniently to studies, diving offers an opportunity. The underwater environment challenges divers’ fluid homeostasis and causes a risk of dehydration with detrimental effects on mental performance and physical work capacity. This is acknowledged by the diving community, but no study to date has systematically examined saturation divers’ hydration status during underwater operations. A study by Wekre et al. examines the outcome of adherence to hydration routines in the Norwegian NORSOK U-100 standard for manned underwater operations on saturation divers’ hydration status before and after daily dives. They concluded that the divers’ routines for fluid intake kept them adequately hydrated, acutely and throughout their period in saturation. The divers’ observance of routines became strikingly visible through the observation that divers working in the dive bell rather than in water scored higher for hydration at the end of their shift than they did when their workday began.

## Changes in muscle physiology induced by changes in environmental conditions

This Research Topic contains information about changes in muscle physiology of different severity but related to changes in ambient temperature and pressure in seven out of the totally eight publications, two of them related to skeletal muscle. The first is a review by Farbu et al. which points to an increased risk of musculoskeletal conditions after cold exposure, manifested as back, neck and shoulder pain. The second is a case report by Yeh et al. reporting acute skeletal muscle damage (rhabdomyolysis) related to high-altitude hypobaric hypoxia.

In contrast to these changes in skeletal muscle physiology in response to extreme environments, the physiologic depression of cardiac function in response to reduction in body core temperature is better known. The study by Schanche et al. and the two studies by Filseth et al. all show reduced cardiac function during core cooling in an experimental model, and the level and severity of depression is closely related to level of temperature reduction. The same studies report that the hypothermia-induced reduction in cardiac muscle function is followed by a substantial increase in vascular smooth muscle activity which consequently increases vascular resistance. In this context, the increased vascular resistance apparently maintained blood pressure to a level in support of maintained spontaneous circulation as cardiac output is reduced to a greater extent than the reduction in blood pressure.

## Therapeutic challenges created during rewarming from experimental hypothermia

The article by Schanche et al. *vs.* the two articles by Filseth et al. clearly uncovers the difference in expected successful rewarming, or not, that depends on the existence of maintained spontaneous cardiac activity, or cardiac arrest, during the hypothermic period. After substantial efforts to improve treatment of accidental hypothermia patient over the past 20 years, mortality is reduced to 28%–35%. But this success rate is closely linked to patients without cardiac arrest, as survival rate of patients in cardiac arrest is much lower. To support global circulation during rewarming from hypothermic cardiac arrest, substantial therapeutic interventions like the use of heart-lung machine (HLM) is essential, as demonstrated in the two last articles. But so far, the use of HLM to rewarm accidental hypothermia patients is based on experience from using this technique in elective heart surgery. Filseth et al. question the similarities between these two patient groups with respect to rewarming by HLM. By comparing the two groups in an experimental setup they show essential differences in circulatory function between groups during cooling, and especially during HLM rewarming. This new information calls for essential new knowledge to optimize the use of extracorporeal rewarming techniques to rescue accidental hypothermia patients in cardiac arrest. In their second article, they compare attempts to do early *vs.* late weaning from HLM and clearly demonstrate the existence of significant post-hypothermic cardiac failure and the need for prolonged extracorporeal circulatory support after normal core temperature is reestablished. Promising, with respect to therapy aimed at increasing survival in hypothermic cardiac arrest patients, was the documentation in the article by Valkov et al. of preserved cerebral autoregulation and blood flow during early start of and continued cardiopulmonary resuscitation at 27°C. Schanche et al. report that rewarming with spontaneous circulatory function may create a hypercoagulable state and a need for antithrombotic intervention. The same authors also report that the increase in vascular smooth muscle activity which created elevated vascular resistance, and thus increased cardiac afterload, remained during rewarming. Combined with the finding of post-hypothermic cardiac dysfunction throughout the rewarming period, this calls for interventions to reduce cardiac afterload. Due to a lack of detailed knowledge of pharmacodynamics and pharmacokinetics of most pharmacologic agents at low core temperature, algorithms advise not to apply drugs at core temperatures <32°C. Thus, the new documentation by Kuzmiszyn et al. that the vasodilator properties of milrinone, amrinone and levosimendan are preserved also below 30°C support their use to achieve afterload reduction during rewarming.

Taken together, this Research Topic presents consequences of exposure to extreme environments, and effects of adaptational interventions. This new information may support the development of written guidelines to improve safety and survival.

